# AGR2, an Endoplasmic Reticulum Protein, Is Secreted into the Gastrointestinal Mucus

**DOI:** 10.1371/journal.pone.0104186

**Published:** 2014-08-11

**Authors:** Joakim H. Bergström, Katarina A. Berg, Ana M. Rodríguez-Piñeiro, Bärbel Stecher, Malin E. V. Johansson, Gunnar C. Hansson

**Affiliations:** 1 Department of Medical Biochemistry, University of Gothenburg, Gothenburg, Sweden; 2 Max von Pettenkofer Institute for Hygiene and Medical Microbiology, LMU Munich, Munich, Germany; Inserm, France

## Abstract

The MUC2 mucin is the major constituent of the two mucus layers in colon. Mice lacking the disulfide isomerase-like protein Agr2 have been shown to be more susceptible to colon inflammation. The Agr2^−/−^ mice have less filled goblet cells and were now shown to have a poorly developed inner colon mucus layer. We could not show AGR2 covalently bound to recombinant MUC2 N- and C-termini as have previously been suggested. We found relatively high concentrations of Agr2 in secreted mucus throughout the murine gastrointestinal tract, suggesting that Agr2 may play extracellular roles. In tissue culture (CHO-K1) cells, AGR2 is normally not secreted. Replacement of the single Cys in AGR2 with Ser (C81S) allowed secretion, suggesting that modification of this Cys might provide a mechanism for circumventing the KTEL endoplasmic reticulum retention signal. In conclusion, these results suggest that AGR2 has both intracellular and extracellular effects in the intestine.

## Introduction

The gastrointestinal tract is protected by mucus that is differently organized along the intestine [1]. In the small intestine there is a single mucus layer, whereas mucus in the stomach and colon is double layered. In colon the inner mucus layer is important for separating bacteria from the epithelium [2]. Defects in this inner mucus layer allow bacteria to get in contact with the epithelial cells, an event which can trigger an inflammatory reaction [3], [4]. The MUC2 mucin forms the skeleton of the intestinal mucus [4]. This gel-forming mucin is thus instrumental in maintaining a functional inner colon mucus layer, and its absence leads to severe colitis and cancer development [2], [5]. This places the goblet cells, which biosynthesize the MUC2 mucin, in the focus of understanding mucus.

Anterior gradient 2 protein (AGR2) is part of the three membered AGR family first identified to be involved in control of the cement gland and brain development of *Xenopus* [6]. In salamanders anterior gradient-related proteins Nag and Mag induce the spectacular regeneration of lost vertebrate limbs [7]. In mammalian breast tissue AGR2 expression is estrogen controlled and regulates mammary epithelial proliferation [8]. Thus AGR proteins have growth promoting effects, a property that is linked to the role of AGR2 in cancer development and metastasis of several tumor types [9]. AGR2 is proposed to act as a pro-oncogene as well as regulating cell proliferation [8], [9]. The *Xenopus* and Salamander mechanisms are due to secreted forms of the AGR-analogues, but the localization of the growth promoting effect in mammals is less understood [9]. Recent studies using recombinant monomeric AGR2 suggested that it could bind on the outside of cells suggesting that AGR2 could have an extracellular effect [10].

Mature AGR2 is a small 154 amino acid protein with a single central Cys residue and a non-conventional endoplasmic reticulum (ER) retention motif (KTEL). The structure of AGR2 was recently revealed and suggested that AGR2 was appearing as a non-covalent dimer in the ER [10]. The Cys residue is part of the CXXS motif that is found in the large family of disulfide isomerases (PDI) and as many members of these proteins also have ER-retention signals, AGR2 have been suggested to act as a PDI and being involved in controlling ER homeostasis [11]. Such a function is supported by the observation that AGR2 can act as a protein disulfide isomerase-like molecule important for MUC2 biosynthesis [12]. To further study AGR2 function, several mice lines (Agr2^−/−^) have been developed [12]–[14]. All of these animals show alterations in different parts of the gastro-intestinal tract.

As AGR2 has been suggested to be involved in the MUC2 mucin biosynthesis we found it essential to further understand the relation between AGR2 and MUC2 [12]. This is of further importance as AGR2 had been genetically linked to inflammatory bowel disease susceptibility [15]. We thus decided to analyze the interaction of AGR2 with the MUC2 mucin in more detail *in vitro* using a cell culture model and *in vivo* in Agr2^−/−^ mice. In the cell culture model we could not confirm direct covalent binding of AGR2 to MUC2. We also observed that AGR2 was secreted when its single Cys was removed (C81S). Lastly, we could show that the intestinal mucus contained a high concentration of secreted Agr2, suggesting extracellular effects among the poorly understood functions of this protein.

## Materials and Methods

### Generation of the pcDNA3.1-AGR2 plasmid

The full length cDNA sequence of the human AGR2 was PCR-amplified from the RZPD clone IRATp970F1215D6 with the primers 5′-CACCATGGAGAAAATTCCAGTGTC-3′ and 5′-CAATTCAGTCTTCAGCAACTTGAGAGC-3′ to introduce a 5′ CACC overhang, needed for directional cloning into the vector pcDNA3.1D/V5-His-TOPO (Life Technologies). Next, a stop codon was introduced in the vector after the AGR2 sequence by site-directed mutagenesis (QuickChange site-directed mutagenesis kit; Stratagene) using the primers 5′- CTCAAGTTGCTGAAGACTGAATTGTAGGGTCAAGACAATTCTGCAG -3′ and 5′-CTGCAGAATTGTCTTGACCCTACAATTCAGTCTTCAGCAACTTGAG-3′ (mismatch is underscored). The PCR was initiated at 95°C for 30 s, followed by 15 cycles (95°C for 30 s, 55°C for 1 min and 10 min at 68°C with a final elongation for 10 min at 68°C.The resulting vector was designated as pcDNA3.1-AGR2 and encoded an untagged version of the human AGR2.

### Subcloning with pcDNA3.1/Zeo(+)

To make stable clones of CHO-psNMUC2-MG [16] and AGR2, the plasmid pcDNA3.1-AGR2 was subcloned to be resistant against Zeocin. The AGR2-containing sequence was excised from the pcDNA3.1-AGR2 vector with the restriction endonucleases BamH1 and Xba1, and ligated in frame into the pcDNA3.1/Zeo(+) vector (Life Technologies) linearized with the same enzymes, generating the expression plasmid named pcDNA3.1/Zeo-AGR2.

### Mutagenesis of pcDNA3.1-AGR2

The mutagenesis was made through PCR reactions, digestion of products and transformation into XL1-Blue super-competent cells according to the QuickChange Site-Directed Mutagenesis Kit (Stratagene). In order to create a DNA-plasmid encoding a Ser instead of a Cys on the 81st amino acid of the protein (AGR2 C81S), the template plasmid pcDNA3.1D-AGR2 was PCR amplified with the forward primer 5′-CATCACTTGGATGAGTCCCCACACAGTCAAGC-3′ and the reverse primer 5′-GCTTGACTGTGTGGGGACTCATCCAAGTGATG-3′. The PCR was initiated at 95°C for 30 seconds, followed by an annealing/elongation phase of 18 cycles (95°C for 30 seconds, 53°C for 1 minute, 6 minutes at 68°C).

To create a DNA-plasmid coding to transcribe a protein with an Asp instead of a Thr residue at position 173 of the protein (AGR2 T173D), the template plasmid pcDNA3.1D-AGR2 was PCR amplified with the forward primer 5′-CAAGTTGCTGAAGGATGAATTGTAGGAGCTCGACAATTCTGC-3′ and the reverse primer 5′-GCAGAATTGTCGAGCTCCTACAATTCATCCTTCAGCAACTTG-3′. The PCR was initialized for 30 seconds at 95°C, followed by an annealing/elongation phase of 18 cycles (95°C for 30 seconds, 52°C for 1 minute, 6 minutes at 68°C).

For construction of the DNA-plasmid encoding a version of AGR2 without the KTEL sequence (AGR2 ΔKTEL), the Lys residue in the terminal KTEL peptide was replaced by a STOP codon. pcDNA3.1D-AGR2 was PCR amplified with the forward primer 5′-CAAGTTGCTGTAGGAGCTCTTGTAGGGTCAAGAC-3′ and the reverse primer 5′-GTCTTGACCCTACAAGAGCTCCTACAGCAACTTG -3′. The PCR conditions were the same as for the AGR2 C81S. All generated plasmids were controlled by nucleotide sequencing after mutagenesis.

### Tissue culture

CHO-K1 cells were obtained from the American Type Culture Collection (CCL-61) and cultured in Iscove's modified Dulbecco's medium (Lonza) containing 10% (v/v) fetal bovine serum. The cell line CHO-pSNMUC2-MG was cultured in the same medium supplemented with G418 (250 µg/mL) [16]. Cells transfected with pcDNA3.1/Zeo-AGR2 were additionally kept in Zeocin (Life Technologies) (250 µg/mL).

### Transfection

Transient transfections were made in CHO-K1 cells or CHO-pSNMUC2-MG cells using to the Lipofectamine 2000. After 48 hours the supernatant was collected. The cells were then incubated 30 min on ice with lysis-buffer containing Complete EDTA-free protease inhibitors (Roche), and collected.

### SDS-PAGE and immunoblotting

For protein separation, samples were mixed with Laemmli sample buffer with or without 200 mM dithiothreitol (DTT), incubated for 5 min at 95°C and separated in a 3–15% polyacrylamide gel by discontinuous SDS-PAGE using a 3% stacking gel [17]. The Precision Protein Standard (Bio-Rad) was used as molecular marker. The separated proteins were Western-blotted onto polyvinylidene difluoride (PVDF) membranes (Immobilon-PSQ Transfer Membrane, Millipore) using a Trans-Blot SD semidry transfer cell (Bio-Rad) at 2.4 mA/cm^2^ (MUC2) or 2.0 mA/cm^2^ (AGR2) for 1 h with blotting buffer (Tris 48 mM, glycine 39 mM, SDS 1.3 mM and 10% or 20% methanol for MUC2 and AGR2 respectively). The membrane was blocked for 1 hour at RT in 5% non-fat milk in PBS with 0.1% Tween 20, and incubated 1 hour at RT or at 4°C overnight with the primary monoclonal antibodies anti-AGR2 (Abnova H00010551-M01, 1∶2000), anti-GFP (Sigma G6539, 1∶4000) or anti-Actin (Millipore MAB1501, 1∶5000) and subsequently with a secondary goat anti-mouse-HRP antibody (Southern Biotech 1∶2000). Membranes were developed with Immobilon Western chemiluminescent HRP substrate (Millipore), imaged using a LAS 4000 analyzer (Fujifilm), and the band intensities measured using the Multi Gauge v3.0 (Fujifilm) software. Band intensities of three replicated blots plotted as histograms using GraphPad Prism v6 (GraphPad, La Jolla, CA).

### Immunohistochemistry

Agr2^−/−^ mice were kindly provided by David Erle (Univ. Calif. San Francisco, CA) [12]. Mice were kept in individually ventilated cages under SPF conditions, sacrificed by CO_2_ inhalation and mouse numbers were reported to the regional authorities (Veterinäramt München, Munich, Germany). These mice and the Agr2^+/+^ (WT) and Agr^+/−^ were housed together and distal colon tissue from these were fixed in Carnoy solution (MethaCarn) to preserve the mucus [2]. Sections were dewaxed using Xylene substitute (Sigma) and hydrated. The antigens were retrieved by microwave heating in 0.01 M citric buffer pH 6.0 and the sections were incubated with the anti-MUC2C3 antiserum 1∶1,000 [2] or anti-GpA Muc2 1∶1,000 dilution [18].

For protein detection in cell lines, the selected clones were fixed with 4% PFA, permeabilized with 0.1% Triton X-100 in PBS, and incubated with the primary antibodies described above for AGR2 and GFP, and with the ER marker anti-calnexin (Abcam ab22595, 5 µg/mL). Alexa Fluor 488, 546 or 555-conjugated goat anti-rat, anti-mouse or anti-rabbit IgG (Life Technologies, 1∶1,000), respectively, were used as secondary antibodies. The slides were counterstained with DAPI (Sigma, 1∶20,000) to visualize the nuclei before mounting with Prolong Gold Antifade reagent (Life Technologies). Pictures were obtained using an upright LSM 700 Axio Examiner 2.1 system with a Plan-Apochromat x20/0.8 DIC objective (Carl Zeiss).

### Collection of murine mucus

For mucus collection, conventional C57BL/6 mice (Taconic, in-house bred) were used with the approval of the local Laboratory Animal Ethics Committee (Gothenburg, Sweden). Mice were anaesthetized with isoflurane and followed by cervical dislocation. Intestinal tissues from 6 mice (3 males and 3 females) ranging from stomach to distal colon were mounted in a horizontal perfusion chamber with a circular opening of 4.9 mm^2^ and luminal mucus was aspirated as described previously [19].

### Collection of human mucus and tissue samples

Human biopsies from control patients (individuals where no abnormalities were reported) were collected after written consent of the patients during routine colonoscopy at Sahlgrenska University Hospital (Gothenburg, Sweden). The study was approved by the Human Research Ethical Committee from the University of Gothenburg (Sweden). Biopsies were mounted in a horizontal perfusion chamber and the secreted mucus aspirated as described [20]. Biopsies were then dismounted and lysed in lysis buffer (50 mM Tris-HCl, pH 8.0, 150 mM NaCl, 1% (v/v) Triton X-100) containing Complete EDTA-free protease inhibitors (Roche), with a Ultra-turrax T8 homogenizer (IKA).

### Proteomics

The relative quantities of Agr2 along the gastrointestinal tract were obtained from a larger proteomics study of the mucus composition in stomach, duodenum, jejunum, ileum, and proximal and distal colon [19]. The molar ratios for Agr2, Muc2, Fcgbp and Clca1 were calculated (in fmol) by the use of the standard peptides from that study, by normalizing the heavy/light ratios for each peptide pair and averaging the results per protein.

## Results

### AGR2 does not bind MUC2, but affects ER morphology

**Figure 1:**
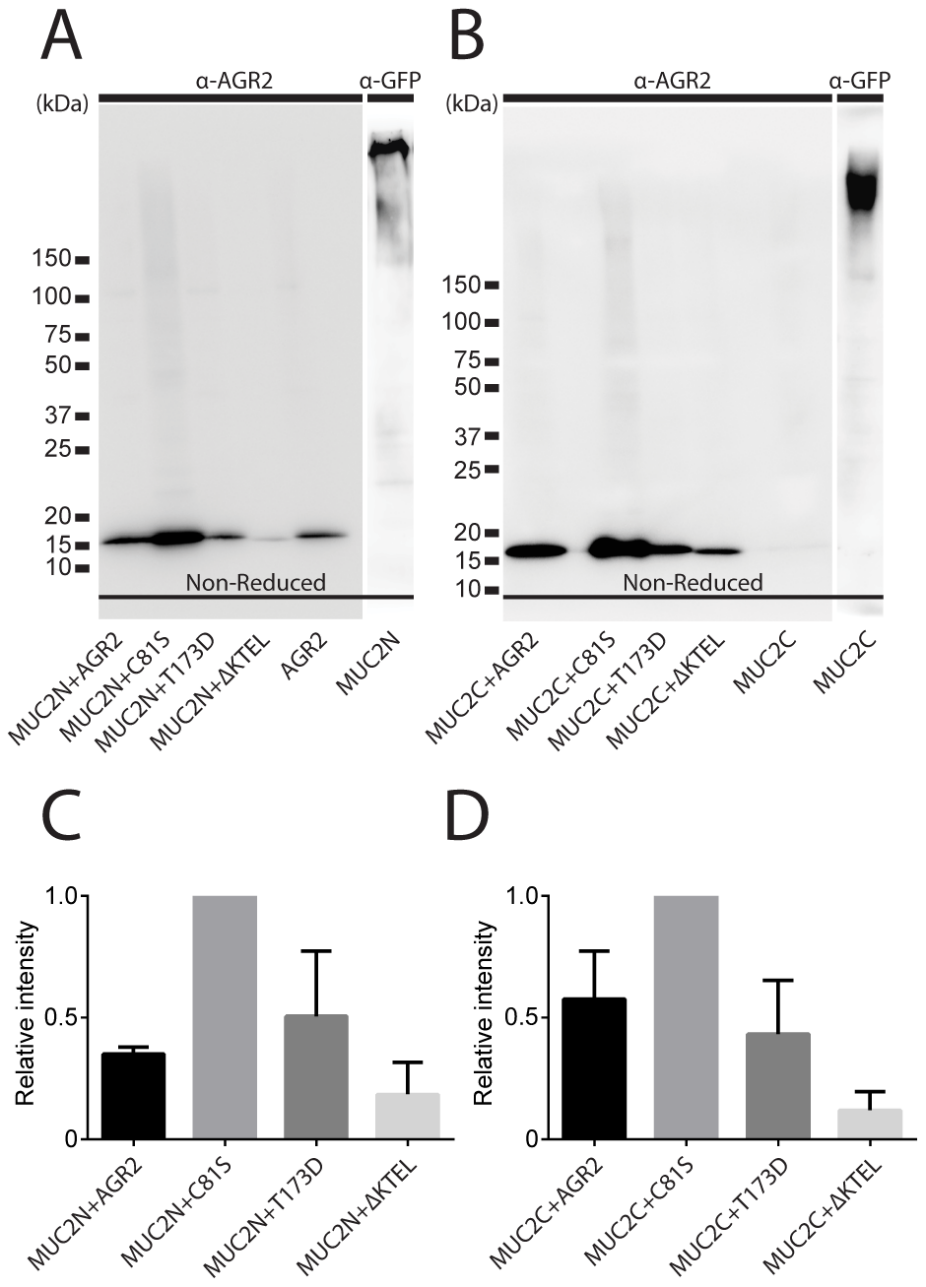
Western blot analysis detecting AGR2 in stably MUC2N- or MUC2C-expressing CHO-K1 cells. The same number of CHO-K1 cells were transiently transfected with equal amounts of expression plasmids coding for human AGR2 and its mutated variants. Equal amount lysates of MUC2N-expressing CHO-K1 cells (A) or MUC2C-expressing CHO-K1 cells (B) had been transfected with the different forms of AGR2, were separated by non-reducing SDS-PAGE and immunoblotted for AGR2. Only a single band at the expected size of AGR2 was observed, and no higher molecular bands were visible indicating that AGR2 did not bind to the MUC2N or MUC2C proteins expressed in the same cell line. Lysates of MUC2N- or MUC2C-expressing CHO-K1 cells immunostained for GFP are shown to the left and right, respectively. Figures are representative of at least 3 replicates. Band intensities of AGR2 normalized to the strongest band (MUC2N-C81S) in the blots of three replicates are plotted as a histogram (C) for MUC2N (correpsonds to gel in A) and (D) for MUC2C (correppnds to gel in B). Error bars represent standard deviation.

AGR2 has been suggested to be covalently attached to the MUC2 mucin [12]. These authors used transient transfection of both MUC2 and AGR2 plasmids and COS-7 or HEK-293T cells. To further address this observation in cells that stably secrete truncated parts of MUC2, we used CHO-K1 cells that are stably expressing the MUC2 N-termini (SNMUC2-MG) or MUC2 C-termini (SMG-MUC2C), respectively [16], [21]. These cell lines were selected as they secrete the two MUC2 parts, something that does not take place during transient transfections as the protein levels are too high to allow proper processing in the endoplasmic reticulum (ER). The CHO-K1 cells were first shown not to express AGR2 endogenously. The two cell lines were then transiently transfected with plasmids encoding either human AGR2 (WT), AGR2 where the Cys at position 81 was replaced by Ser (C81S), AGR2 sequences where the C-terminal ER retention signal KTEL of AGR2 had been altered to the canonical ER retention signal KDEL (T173D), or AGR2 lacking the entire KTEL retention signal (ΔKTEL). Cell lysates from these transiently transfected cells were separated on non-reducing gels allowing the MUC2N trimer and MUC2C dimer to enter the gel. The Western blots were immunostained with an anti-AGR2 antibody or with anti-GFP for the MUC2N and MUC2C GFP fusion products (Fig. 1). In these gels, we could not observe any shifts of AGR2-containing bands larger than the AGR2 monomer. This suggests that in our cell model, AGR2 and MUC2 do not covalently interact.

Further attempts to demonstrate an AGR2-MUC2 interaction were made by co-immunoprecipitation using either anti-AGR2 or anti-MUC2 antibodies on CHO-K1 cells permanently or transiently expressing MUC2N or MUC2C and transfected with AGR2. None of these experiments showed any indication of a non-reducible binding between AGR2 and MUC2 (results not shown). Therefore we concluded that most likely there is no covalent, disulfide bonded interaction between AGR2 and MUC2 in the cells used in this study.

**Figure 2:**
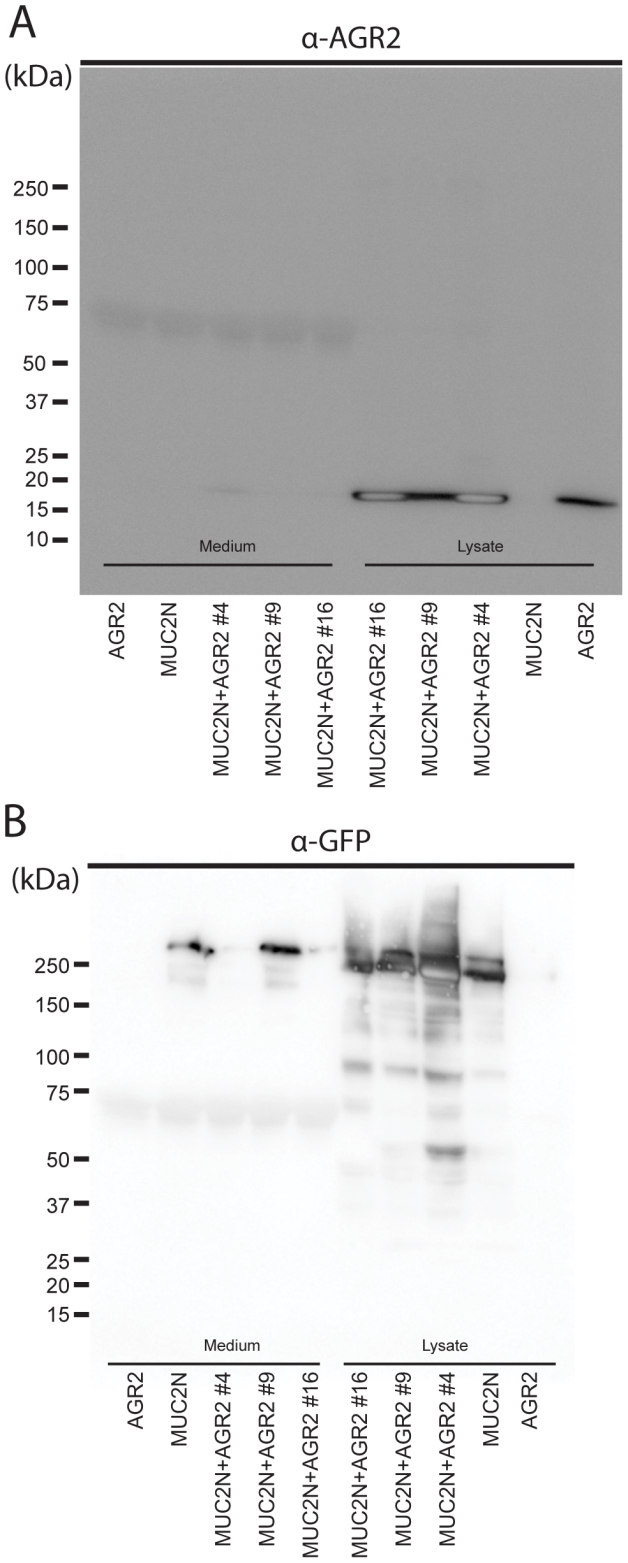
Western blot analysis of CHO-K1 cells stably expressing MUC2N and stably variable levels of AGR2. (A) Spent culture media and lysates normalized to similar number of cells were immunoblotted using an anti-AGR2 antibody after reducing SDS-PAGE. Notice that bands blighted in the center have the highest concentration of AGR2. The stable clones showed varying amounts of AGR2 (#9<#16<#4). Band intensities of AGR2 normalized to the strongest band (clone #4) in the blots of three replicates are ahown as a histogram in Fig. S1. (B) Immunoblot with an anti-GFP antibody detecting the MUC2N protein using the same media and lysates as in (A). In the lysates MUC2 was largely found as the not fully glycosylated form (lower band, slightly below 250 kDa kDa). The mature glycosylated form of MUC2 (higher band, above 250 kDa kDa) was found in the media from the control sample (MUC2N) and clone #9 (MUC2N AGR2 #9). Figure is representative of at least 3 replicates.

As we could not confirm direct interaction between AGR2 and MUC2, we further studied the effect of AGR2 in MUC2 secretion. The CHO-K1 cell line stably expressing MUC2N was transfected with WT AGR2, and stable clones with different AGR2 expression levels were selected. The expression levels of AGR2 were first analyzed by SDS-PAGE and Western blot (Fig. 2A). The clone designated #9 had the lowest intracellular level of AGR2, whereas clone #4 had the highest and clone #16 displayed an intermediate AGR2 expression level. WT AGR2 was not secreted into the culture medium for any of these clones, except that a small amount of AGR2 could be observed in the clone (#4) with the highest AGR2 expression level. When the media and lysates were probed for the stably expressed MUC2N by detecting its GFP-tag, clone #9 showed secretion of MUC2N as was the case for the parent non-AGR2-transfected CHO-K1 cell line (Fig. 2B). None of the two high-expressing clones (#4 and #16) secreted any MUC2N, although all of them showed intracellular MUC2N. This suggested, in contrast to our hypothesis, that AGR2 did not promote folding, maturation, or secretion of MUC2. Instead it suggested that AGR2 retained MUC2 in the cells.

**Figure 3:**
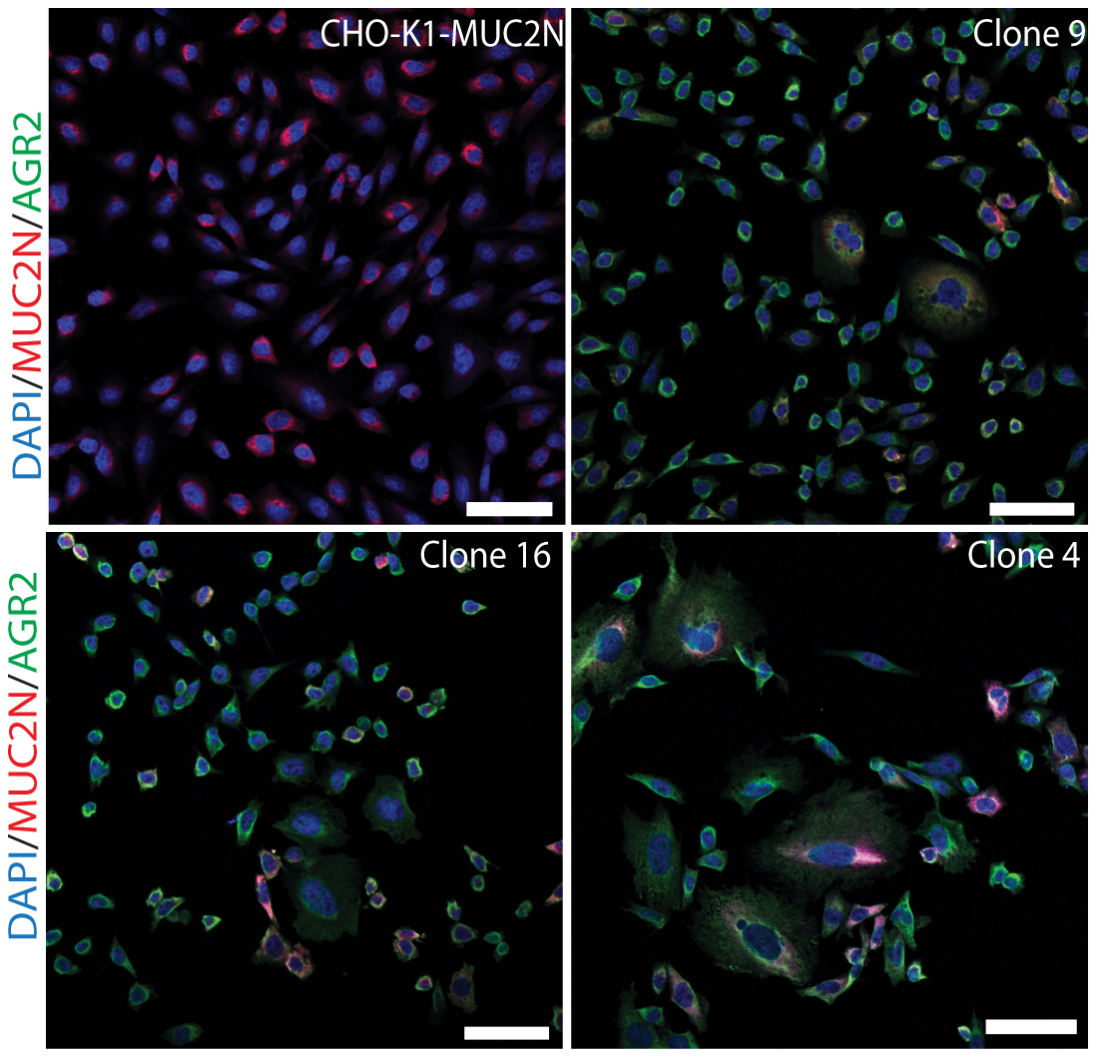
AGR2 alters the morphology of the ER. Immunostaining of the stable MUC2N and AGR2 expressing CHO-K1 cells (see Fig. 2 2). Parent CHO-K1 cells stably expressing various amounts of AGR2 (green) show an increased accumulation of MUC2N and a more severely altered cell morphology depending on the level of AGR2 (clone #9<#16<#4, see Fig. 2 2). Nuclei are stained in blue with DAPI. Scale bar: 50 µm. For quantitative comparisons, see Fig. 2 2. Figures are representative of at least 3 replicates.

The clones #4, #9 and #16, expressing MUC2N and different amounts of AGR2 were studied by immunofluorescence (Fig. 3) of MUC2N (red) and AGR2 (green). The levels of AGR2 varied from lowest to highest in the order #9<#16#<#4, as shown by Western blot (Fig. 2A). In relation to AGR2, the clones displayed increased and aberrant intracellular staining of MUC2N and especially clone #4 showed cells with an increased nuclear volume. As MUC2N accumulates in the ER, this observation suggests that AGR2 affected both ER structure and function. These results also made it less likely that AGR2 have any effect in promoting MUC2 maturation and secretion and suggest instead a more general effect on ER function.

### Agr2^−/−^ mice lack the colonic inner mucus layer

**Figure 4:**
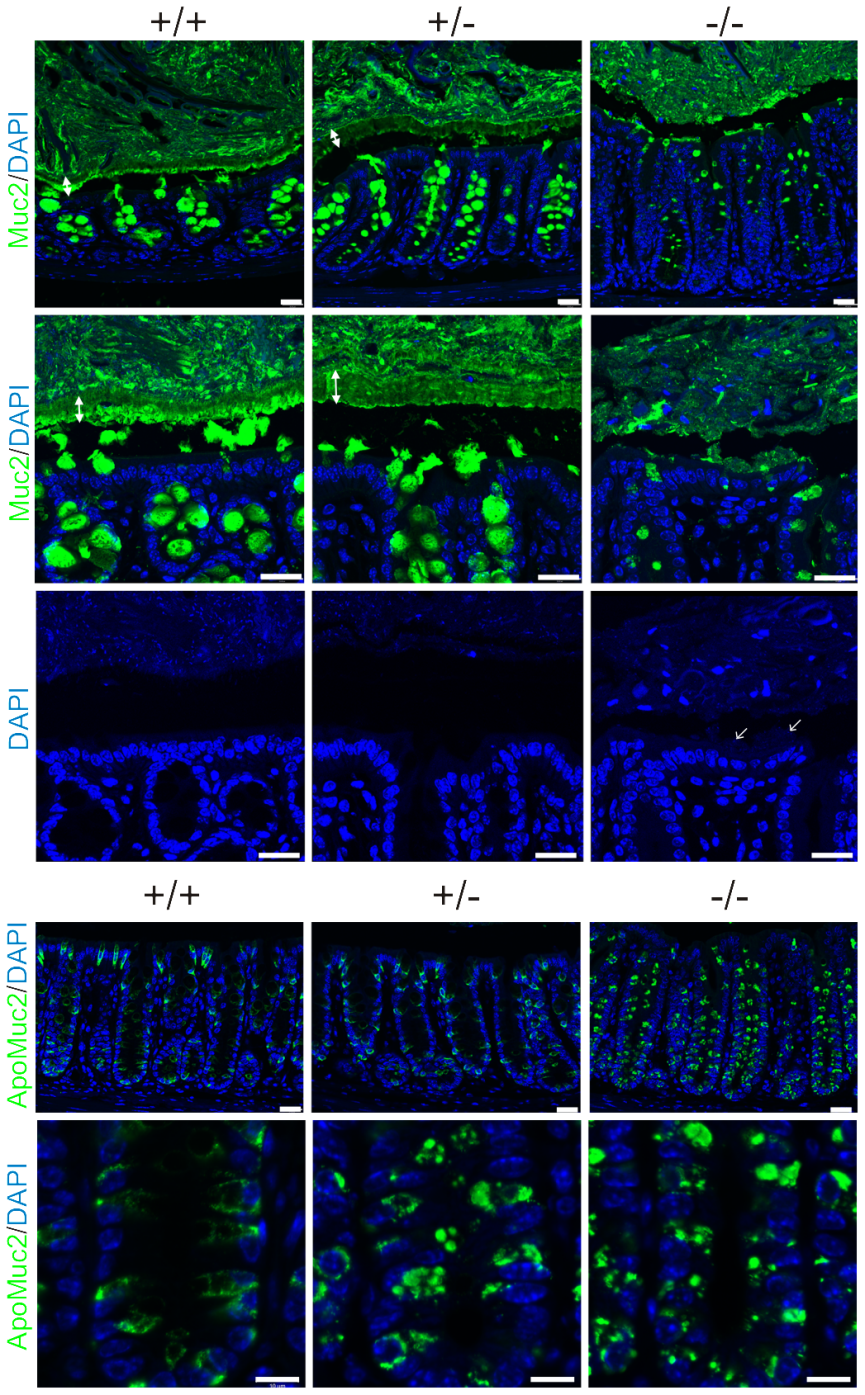
Immunostaining of sections from distal colon from Agr2^+/+^ (+/+) n = 5, Agr2^+/−^ (+/−) n = 2, and Agr2^−/−^ (−/−) n = 3 mice, two sections of each mouse were stained and examined. Two sections each of all Agr2^−/−^ animals are shown in Fig. S2. The two upper panels display two sections each for the mature Muc2 (detected with anti-MUC2C3 antiserum, green) using a glycosylation independent antiserum (the gap between the inner mucus layer and the epithelium is due to shrinking during processing). A decreased amount of mature Muc2 stored in the mucin granules can be observed in the Agr2^−/−^ mice. The middle panel with the DAPI channel alone most likely show bacteria stained as the smallest dots close to the epithelium in the Agr2^−/−^ mice as indicated by arrows. The larger blue dots further away from the epithelium are shed cells or fecal content. The two lower panels show sections at two magnifications stained with an antiserum against the nonO-glycosylated Muc2 precursor (anti-gpA antiserum, green) found in the ER. A more fragmented ER with accumulation of immature Muc2 is observed in the Agr2^−/−^ mice. DNA stained with DAPI (blue). Scale bar 25 µm for the top three panels, scale bar 10 µm for the lowest panel.

The distal colon of Agr2^+/+^, Agr2^+/−^, and Agr2^−/−^ mice was fixed in Carnoy's solution and stained for mature Muc2 (Fig. 4, two upper panels). In the Agr2^+/+^ and Agr2^+/−^ animals, a typical inner mucus layer was observed that lacked bacteria (stained as small blue dots). However, in the Agr2^−/−^ mice this inner layer was almost absent and DNA-staining most likely representing bacteria could be found in all mucus and sometimes in contact with the epithelial cells as shown by arrows in Fig. 4, middle panel. Staining for Muc2 showed filled goblet cells, although at a lower level in Agr2^−/−^ than in the Agr2^+/−^ and Agr2^+/+^ animals.

When the colonic tissues were stained with an antiserum against the non-O-glycosylated form of Muc2 (the ER-localized precursor of the molecule), the Agr2^+/+^ tissue was stained as typical ER (Fig. 4, lower two panels). However, the patterns in the Agr2^−/−^ tissues were more intense and considerably more punctuated resembling a more fragmented ER. Together this shows that Muc2 is formed in the Agr2^−/−^ mice, but the amounts eventually processed and secreted might be insufficient to form a functional mucus layer.

### Agr2 is secreted and found abundantly in the gastrointestinal mucus

**Figure 5:**
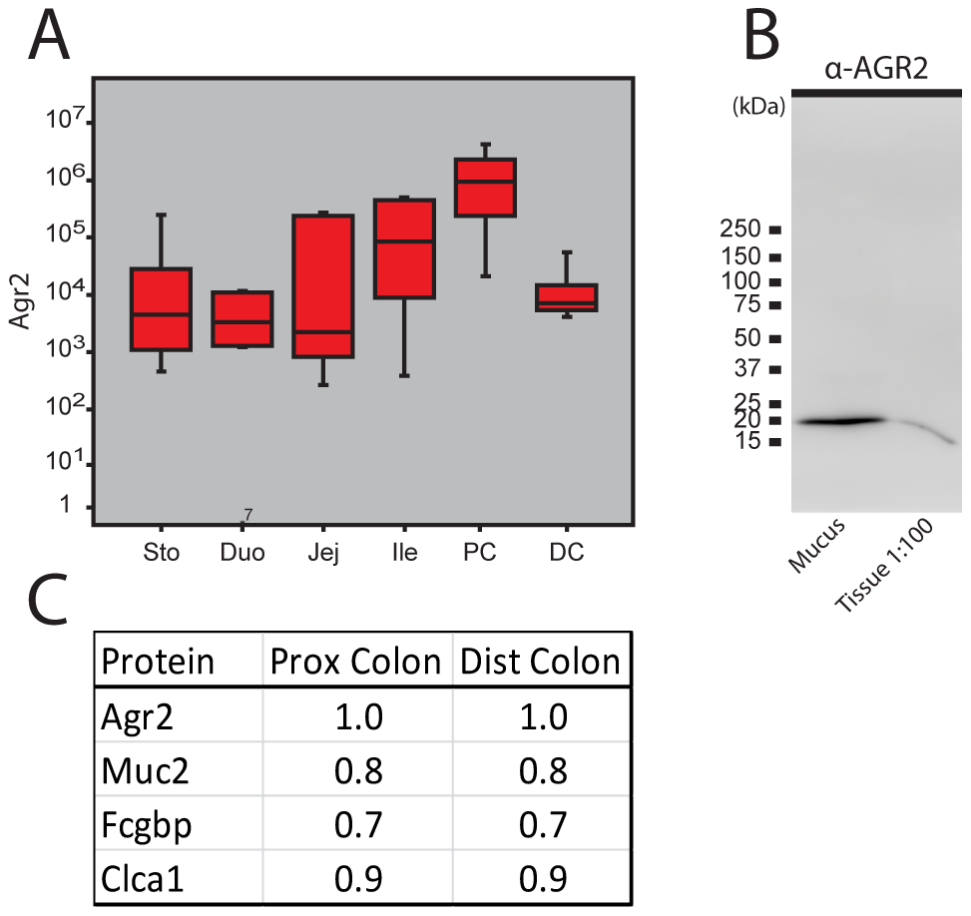
Agr2 is a component of the protective mucus layer in the whole gastrointestinal tract. (A) Boxplot of the relative levels (logarithm of the normalized intensities) of Agr2 in the mucus along the mouse gastrointestinal tract as revealed by proteomics. Sto: stomach; Duo: duodenum; Jej: jejunum; Ile: Ileum; PC: proximal colon; DC: distal colon. (B) Immunoblot detecting AGR2 in mucus from human colonic biopsies mounted in a horizontal perfusion hamber. The first lane (mucus) contains the collected secreted mucus. The second lane (lysate) shows the AGR2 remaining in the biopsy after mucus collection (diluted 1∶100). (C) Molar ratios between Agr2, Muc2, Fcgbp and Clca1 in proximal and distal colonic murine mucus.

In studies of the mouse colonic mucus by proteomics Agr2 was observed to be a relatively abundant protein [2], [22]. We have now expanded our mucus proteomic studies to the whole gastro-intestinal tract and observed that Agr2 was present in the mucus of all parts of the digestive tract reaching the highest concentrations in the proximal colon (Fig. 5A). To further confirm the extracellular localization of also the human AGR2, colonic mucus from human biopsies was carefully collected after mounting it in a horizontal Ussing-type chamber, and both the collected mucus and the remaining intestinal tissue were analyzed by PAGE and Western blot. An AGR2 band at about 18 kDa was found in the collected mucus as well as in the colon tissue (Fig. 5B). It can thus be concluded that both the human AGR2 and the murine Agr2 are not only resident ER proteins, but they are also present in significant amounts in the secreted gastrointestinal mucus.

The molar amounts of Agr2 in mouse mucus were further analyzed by proteomics in relation to known abundant mucus proteins (Muc2, Fcgbp, and Clca1) by adding internal heavy peptide standards during mass spectrometry analyses of the collected mucus [19]. The molar ratios for these four proteins were relatively similar (Fig. 5C). In fact, as Agr2 was suggested to be the most abundant further support the idea that Agr2 is secreted and may have also extracellular functions.

### AGR2 is retained in the ER by its single Cys amino acid and the KTEL sequence

**Figure 6:**
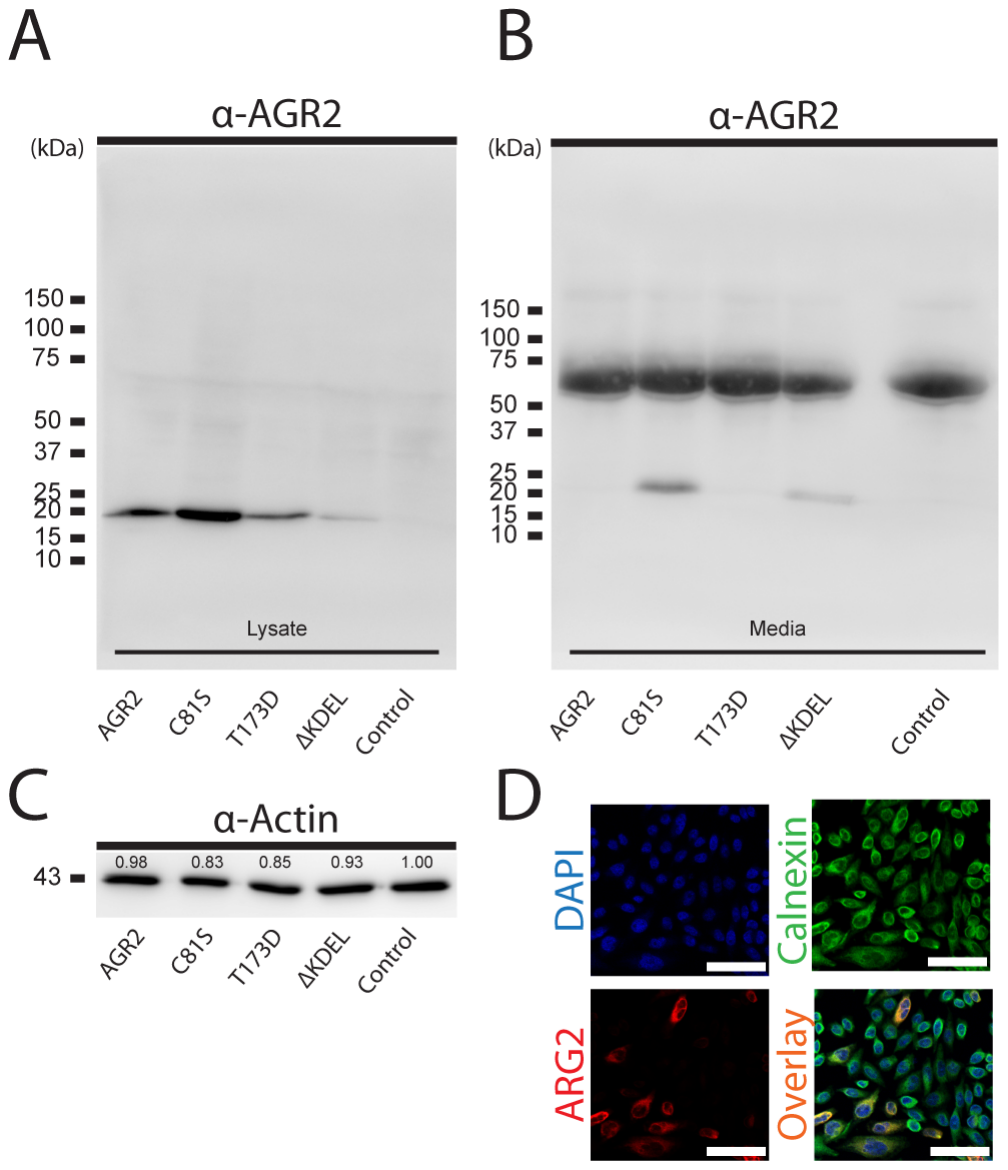
AGR2 detection in CHO-K1 cells transfected with plasmids encoding the human AGR2 and mutated versions. (A) Cell lysate and (B) spent culture media of the transfected cells separated on a reducing SDS-PAGE and Western blotted. Band intensities of AGR2 normalized to the strongest band (C81S) in the blots of three replicates in (A) and (B) are ahown as a histogram in Fig. S3. All the AGR2 forms were found in the lysate at the expected size. The truncated AGR2 (ΔKTEL) and the C81S were secreted out into the media. C. Loading control for panel A. showing α-actin expression in corresponding lanes. Band intensities normalized to the strongest band in the blot is noted in the corresponding lane. (D) AGR2 transfected CHO-K1 cells immunostained for AGR2 (red) show ER localization, cells co-stained with the ER-marker Calnexin (green).

The murine Agr2 and the human AGR2 were found in considerable amounts in the intestinal mucus, despite the presence of the non-typical ER retention signal KTEL in their C-termini. AGR2 expressed in CHO-K1 cells was also retained in the ER and not secreted (see Fig. 6D). To clarify these discrepancies, CKO-K1 cells were transiently transfected with plasmids encoding human WT AGR2, AGR2 with its Cys81 replaced by Ser (C81S), AGR2 with its C-terminal KTEL altered to KDEL (T173D), or AGR2 without its C-terminal KTEL (ΔKTEL). Proteins from the cell lysates were separated by reducing PAGE and AGR2 detected by Western blot (Fig. 6A–B). All the AGR2 protein variants were detected in the cell lysates (Fig. 6). However, only the C81S and ΔKTEL AGR2 forms were found in the spent culture media. That the ΔKTEL was not retained has been shown before and was thus expected [23]. However, that its single Cys was involved in controlling ER exit has not been observed previously. The results suggest a specific functional coupling between the ER retaining KTEL signal and the single free Cys for AGR2 retention.

## Discussion

The importance of AGR2 for a proper mucin and mucus formation was proven previously [12]. These Agr2^−/−^ mice showed despite this little colonic inflammation, but DSS (Dextran Sodium Sulfate) challenge gave a more severe inflammatory phenotype than in control animals. The increased DSS susceptibility of Agr2^−/−^ mice were further illustrated and explained by our observed lack of a typical inner mucus layer. Our previous studies have shown that a well-functional inner colon mucus layer is necessary for separating the bacteria from the epithelial cells and that bacteria in massive contact with the epithelium trigger inflammation [2], [3], [24]. Although no typical inner mucus layer was observed in the Agr2^−/−^ mice, these mice still secreted Muc2 mucin as observed by immunostaining. This remaining secretion did not form a proper mucus layer, but still was sufficient to keep the crypts free of bacteria. No bacteria were found inside the epithelial cells as observed for the Muc2^−/−^ mice. The Agr2^−/−^ goblet cell granulae was less filled with mature Muc2 compared to WT mice, but there seemed to be sufficiently mucin secreted to maintain a steady mucus flow to move bacteria away from the epithelial cells. However, further challenging with DSS suggested that the Agr2^−/−^ mice had more difficulties in handling bacterial stress [12], something that is well in line with a less developed inner mucus layer.

In this work, we could not confirm the previous observation of a direct binding of the AGR2 and the MUC2 N- or C-terminal protein fragments [12]. Although the MUC2 plasmids used were the same as in the previous study, a reason for this discrepancy could be the different cell lines used. In this case, we employed CHO-K1 cells stably secreting the MUC2 proteins, as we know that transient transfection of the corresponding plasmids induce such a high expression level that most of the MUC2 proteins (especially the N-terminal end) accumulates in the ER. It is only in stable expressing cell lines with lower mRNA and protein levels that secretion is observed. The high number of Cys in MUC2 could be an attractive explanation for why AGR2 was previously observed to bind covalently to MUC2, as this could bind free Cys in MUC2. In the previous study [12], the binding between AGR2 and MUC2 was only observed intracellular and might be due to non-specific binding due to overexpression. The domain organization and the localization of the Cys in MUC2 is almost identical to that of the von Willebrand factor [25], a protein that evolved from metazoan mucin-like proteins [26]. However, AGR2 is not expressed in endothelial cells (http://www.proteinatlas.org) that synthesize the von Willebrand factor, suggesting that AGR2 is not necessary for maturation of this molecule. This further argues against AGR2 being absolutely necessary for MUC2 biosynthesis and secretion.

Several Agr2^−/−^ mouse lines have been generated and found to have variable defects in different organs [12]–[14]. In particular, Park *et al*. showed an almost complete absence of goblet cells in the small and large intestine [12], something that we could not observe in this study using the same Agr2^−/−^ mouse strain. A number of reasons for this difference could be envisioned as different bacteria found in the mouse housing facilities, different ages of the mice, different anti-Muc2 antibodies, or different levels of inflammation. It is well known that inflammation can trigger goblet cell secretion and emptying of the goblet cells and by this give the impression of lost goblet cells. Zhao *et al.* also observed less intestinal goblet cells in another Agr2^−/−^ mice line, using both a germ-line knock-out as wells as an inducible deletion [13]. They also observed atypical and abnormally filled Paneth cells. The Paneth cells produce antibacterial peptides, lysozyme and are also producing and secreting MUC2 mucin. Thus AGR2 not only triggers goblet cell emptying, but also accumulation in Paneth cell granulae further arguing for AGR2 having a more complex function that are dependent on cell type. These mice displayed both severe spontaneous colitis and ileitis, whereas the ones from Park *et al*. required a DSS challenge to present a more severe phenotype than the WT controls. Finally, Gupta *et al*. studied the stomach of yet another Agr2^−/−^ mouse line and demonstrated a remarkable mucosa hyperplasia of the glandular stomach, giving rise to stomach outlet obstruction [14]. From these results one can conclude that lack of Agr2 leads to different outcome of different cell types, something that obscures an understanding of AGR2 function.

AGR2 has a large effect on the ER as has been suggested in a number of articles [11]–[13], [23]. However, the mechanism and functional effect of AGR2 is not understood, but AGR2 have both morphological and functional effects on the ER. We could also show that loss of Agr2 leads to a fragmented ER and that overexpression gives rise to an enlarged ER. Overexpressing AGR2 in CHO-K1 cells already secreting MUC2N caused decreased MUC2N secretion suggesting that AGR2 did not facilitate MUC2N processing.

The function of AGR2 in the ER is still not understood. The structure of AGR2 recently revealed that AGR2 forms a non-covalent dimer in solution [10]. This dimer is formed based on non-covalent charge interactions and not by any disulfide-bond interactions. This dimer is unstable and requires artificial cross-linking to be observed something that explains why we have never observed AGR2 dimers [27]. This non-covalent dimeric form of AGR2 might be important for its ER function [27]. Interestingly, the structural work also suggested that cell binding only required monomeric AGR2 [10], supporting the idea that AGR2 may also have extracellular functions.

That the AGR2 C-terminal KTEL ER retention motif has a special function that is different from the more typical KDEL has already been suggested [23]. AGR2 expression in cancer cells induces the expression of amphiregulin (AREG) and in intestinal IEC-6 cells the transcription factor CDX2 [23], [28]. Interestingly, replacing the KTEL with KDEL made AGR2 incapable of inducing CDX2 expression although AGR2 were still retained in the ER [23]. The importance of the KTEL sequence is also illustrated by its presence in the anterior gradient-like proteins Nag and Mag, which are involved in vertebrate limb generation [7]. Interestingly, these proteins also carry the single Cys residue, exactly as in AGR2. Our observation that AGR2 was not retained in CHO-K1 cells when this Cys was replaced by Ser suggests that the Cys could be coupled to ER retention. One can speculate that the KTEL mediated ER retention is linked to the presence of a free Cys and that modification or binding of this Cys to another molecule allow AGR2 to exit from the ER. Of course other alternatives, as for example misfolding AGR2, may explain secretion of the C81S. Studies of the role of a single Cys combined with a C-terminal KDEL sequence requires further studies.

Our studies of the gastrointestinal tract show that Agr2 is present in mucus throughout the entire gastrointestinal tract. The role of Agr2 in the mucus is not understood, but that its molar levels are similar or maybe even higher than the Muc2 mucin strongly suggest an extracellular function. This hypothesis is supported by AGR2 involvement in cancer progression or in normal mammary gland lobular development as controlling cellular development and regeneration [8], [9]. However, this function is probably complex and cell specific, as the absence of Agr2 caused hyperplasia of specific cells in the glandular stomach and for the Paneth cells, but not for other intestinal cells [13], [14]. The structural studies also suggest that AGR2 have yet unknown cell binding sites [10].

To conclude, AGR2 has complex effects on the ER morphology and its single Cys amino acid may be involved in the control of AGR2 retention in the ER. The high concentration of Agr2 in the gastrointestinal mucus suggests extracellular functions.

## Supporting Information

Figure S1 **Histogram of band intensities corresponding to immunoblotted gel in** **Fig 2A** **.** Intensities were measured from three replicate blots and normalized to the strongest band in the blot (clone #4). Annotations a in legend to Fig. 2. Error bars represent standard deviation.
(TIF)

Figure S2 **Immunostaining of distal colon of Agr2^−/−^ mice.** Pictures show three different animals at two separate locations. Green panel represent mature Muc2 (stained with antiserum anti-MUC2C3) and the blue panel to the right represent DNA staining (DAPI). In all the Agr2^−/−^ sections, small blue dots and rods likely represent bacteria found throughout the sparsely mucus. No pronounced inner mucus layer devoid of bacteria can be seen as found for the Agr2^+/+^ and Agr2^+/−^ animals (Fig 4). The gap between the mucus and epithelia is largely due to shrinkage during fixation. Scale bar 10 µm.
(TIF)

Figure S3 **Histogram corresponding to immunoblotted gels reproduced in** **Fig. 6A** **and** **Fig. 6B** **.** Band intensities measured from three replicate blots and normalized to the strongest band (C81S). Annotation as in legend to Fig. 6. Error bars represent standard deviation.
(TIF)
