# Supplementary figures and images for: AGR2, an Endoplasmic Reticulum Protein, Is Secreted into the Gastrointestinal Mucus
Source: PLoS One. 2014 Aug 11;9(8):e104186. doi: 10.1371/journal.pone.0104186 (PMC4128659; doi:10.1371/journal.pone.0104186)

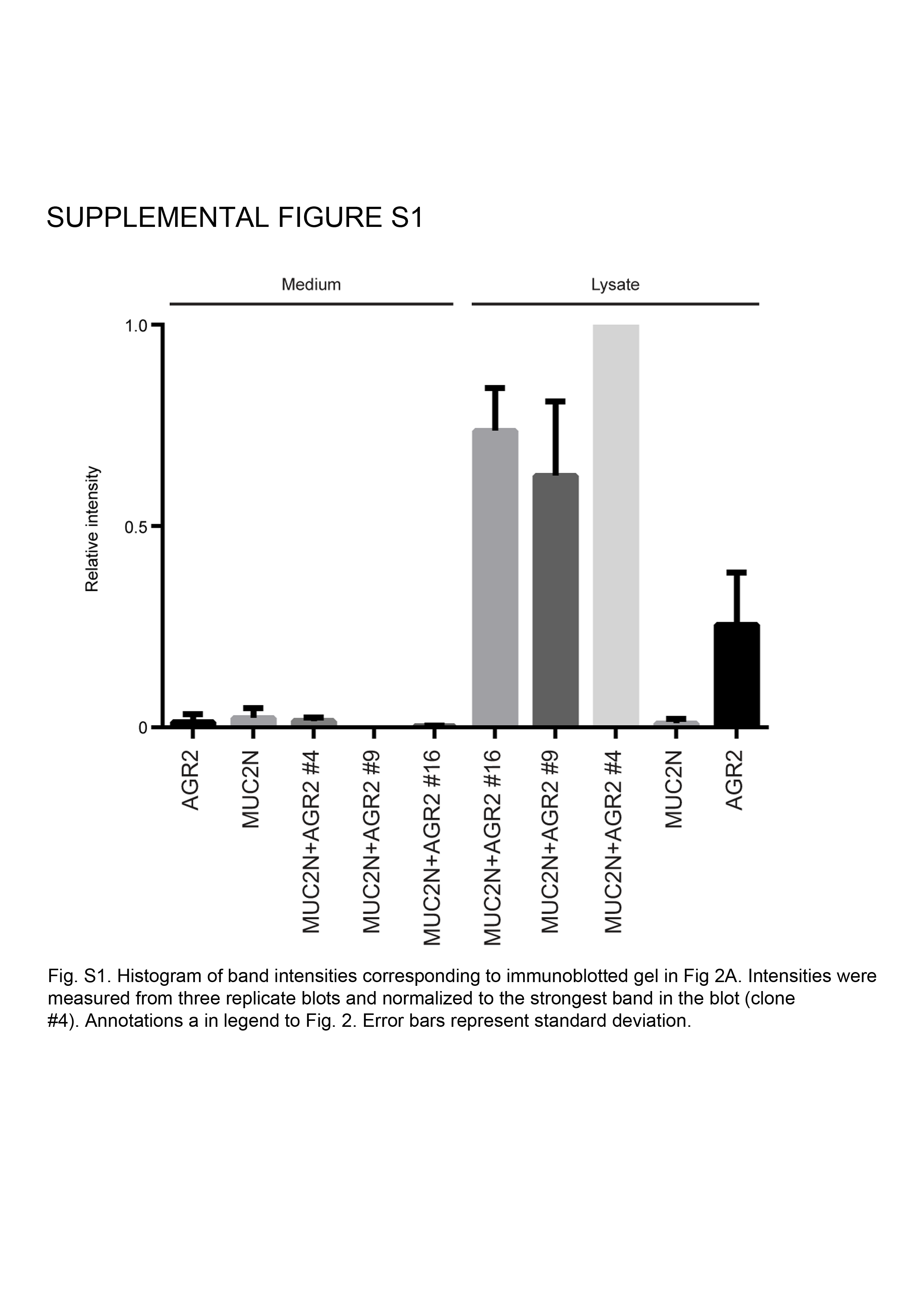

Supplement: Figure S1 — Histogram of band intensities corresponding to immunoblotted gel in Fig 2A . Intensities were measured from three replicate blots and normalized to the strongest band in the blot (clone #4). Annotations a in legend to Fig. 2. Error bars represent standard deviation. (TIF) [file pone.0104186.s001.tif]

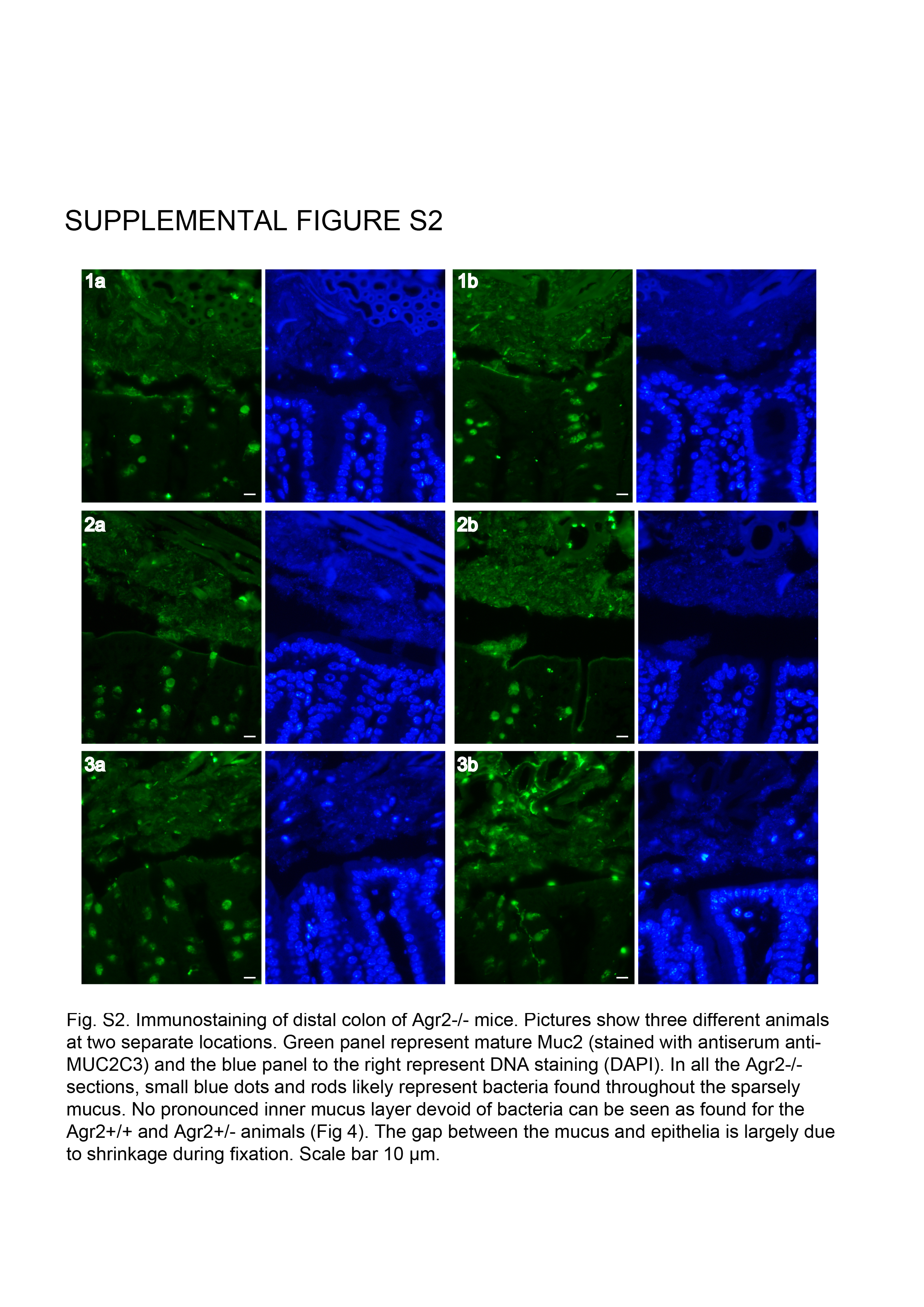

Supplement: Figure S2 — Immunostaining of distal colon of Agr2−/− mice. Pictures show three different animals at two separate locations. Green panel represent mature Muc2 (stained with antiserum anti-MUC2C3) and the blue panel to the right represent DNA staining (DAPI). In all the Agr2−/− sections, small blue dots and rods likely represent bacteria found throughout the sparsely mucus. No pronounced inner mucus layer devoid of bacteria can be seen as found for the Agr2+/+ and Agr2+/− animals (Fig 4). The gap between the mucus and epithelia is largely due to shrinkage during fixation. Scale bar 10 µm. (TIF) [file pone.0104186.s002.tif]

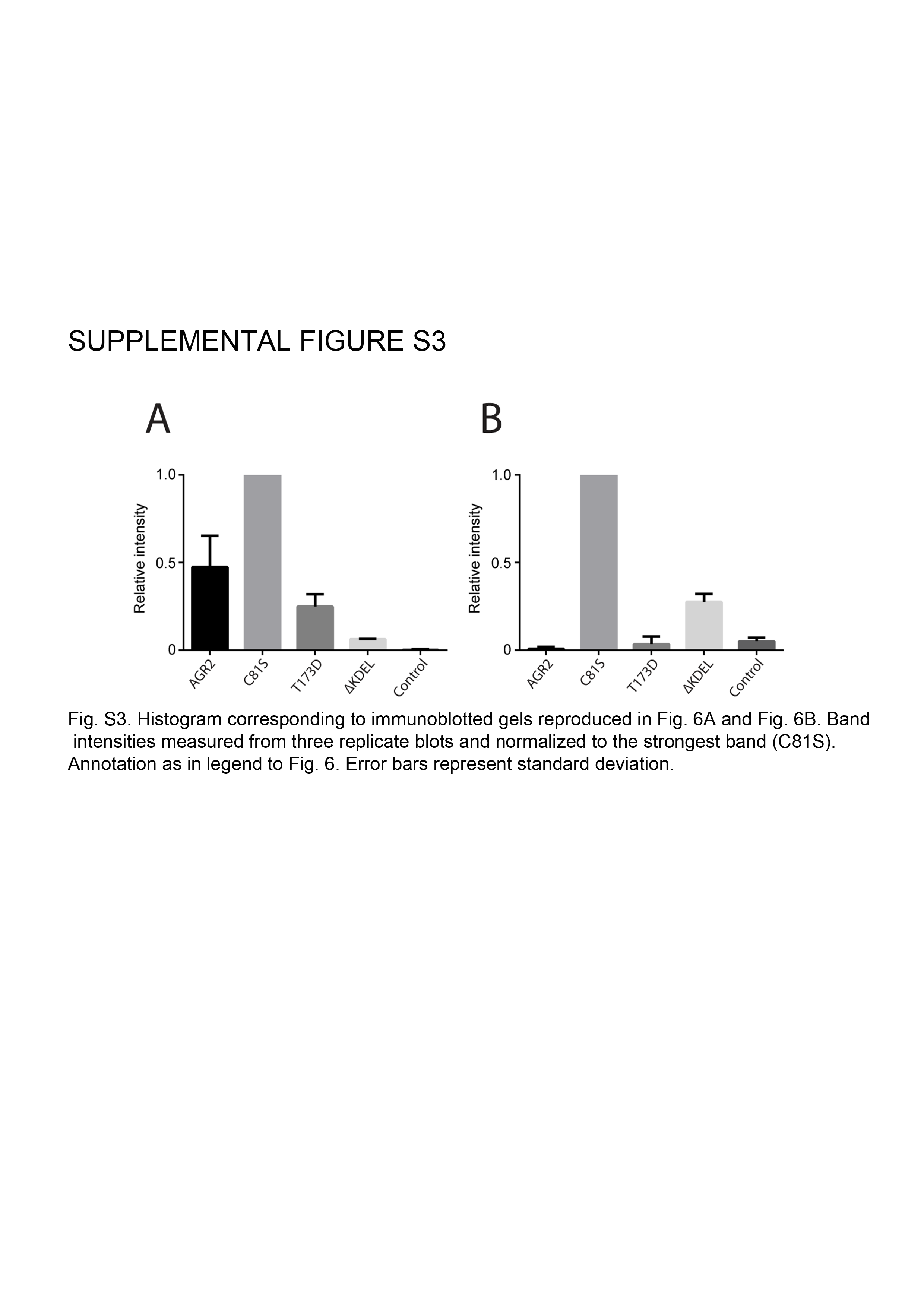

Supplement: Figure S3 — Histogram corresponding to immunoblotted gels reproduced in Fig. 6A and Fig. 6B . Band intensities measured from three replicate blots and normalized to the strongest band (C81S). Annotation as in legend to Fig. 6. Error bars represent standard deviation. (TIF) [file pone.0104186.s003.tif]
